# Fullerene Derivative with Flexible Alkyl Chain for Efficient Tin-Based Perovskite Solar Cells

**DOI:** 10.3390/nano12030532

**Published:** 2022-02-03

**Authors:** Chengbo Tian, Chao Sun, Jingfu Chen, Peiquan Song, Enlong Hou, Peng Xu, Yuming Liang, Panpan Yang, Jiefeng Luo, Liqiang Xie, Zhanhua Wei

**Affiliations:** Xiamen Key Laboratory of Optoelectronic Materials and Advanced Manufacturing, Department of Materials, Institute of Luminescent Materials and Information Displays, College of Materials Science & Engineering, Huaqiao University, Xiamen 361021, China; sunchao@stu.hqu.edu.cn (C.S.); chenjf@stu.hqu.edu.cn (J.C.); reinhardtspq@stu.hqu.edu.cn (P.S.); hquhel@163.com (E.H.); xupengpatrick@stu.hqu.edu.cn (P.X.); lymym@foxmail.com (Y.L.); panpanyang@stu.hqu.edu.cn (P.Y.); jfluo@stu.hqu.edu.cn (J.L.); lqxie@hqu.edu.cn (L.X.)

**Keywords:** functional fullerene, tin-based perovskite solar cells, electron-transporting, stability

## Abstract

Fullerene derivatives are considered excellent materials for the extraction and transportation of electrons in the production of efficient tin-based perovskite solar cells (TPSCs). However, it is not clear how the molecular structure of fullerene derivatives affects the efficiency and stability of TPSCs. In this study, the effects of fullerene derivatives, (6,6)-phenyl-C_61_-butyric acid hexyl ester (PCBH) and (6,6)-phenyl-C_61_-butyric acid methyl ester (PCBM), with different functional groups, on photovoltaic performance were investigated. The flexible alkyl chain of PCBH effectively improved the film morphology and stability, the electron extraction and transport capabilities, and the interface contact of fullerene and perovskite. As a result, the PCBH-based TPSC yielded a higher efficiency, of 9.21%, than the PCBM-based devices (7.54%). More importantly, the PCBH-based films exhibited higher stability and effectively suppressed the oxidation of Sn^2+^ by inhibiting oxygen permeation. Therefore, the PCBH-based devices exhibited significantly enhanced stability. This result indicates that optimizing the functional group of fullerene derivatives is crucial for improving the efficiency and stability of TPSCs.

## 1. Introduction

Tin-based halide perovskite materials have attracted much attention due to their low toxicity, suitable band gap, and their rapid improvement of the performance of photovoltaic devices [1,2,3,4,5]. Tin-based perovskite solar cells (TPSCs) show significant potential owing to their planar device structure, low-temperature solution fabrication process, and potential applications in flexible and tandem devices [6,7,8,9,10,11,12,13]. Fullerenes and their derivatives are widely used as electron transport layers (ETL) in TPSCs. Therefore, as the major ETLs in TPSCs, fullerene derivatives deserve more attention and research. Although many studies are devoted to exploring novel fullerene derivatives, such as ETLs [14,15,16,17,18,19,20], dopants [21,22,23,24,25], or modified layers [26,27,28] to enhance the performance of lead-based perovskite solar cells (LPSCs), few studies have explored the application of new fullerene materials in TPSCs.

Currently, the most commonly used fullerene materials in TPSCs are still (6,6)-phenyl-C_61_-butyric acid methyl ester (PCBM) and C_60_ [7,28]. It is important to realize that improving the stability of fullerene ETLs is critical to the efficiency and stability of TPSCs. As previously reported, PCBM-based ETLs usually display a certain degree of self-aggregation that decreases the long-term stability of LPSCs. To address this issue, Xie et al. mixed two different functional fullerenes to regulate their noncovalent intermolecular interactions and packing mode, thereby improving their device’s efficiency and stability [29]. Recently, we modulated the intermolecular interactions of a fullerene derivative (PCBB) by changing the functional group, which also effectively suppressed the self-aggregation of fullerene molecules [30]. Therefore, designing new fullerene derivatives to regulate intermolecular interactions is an effective strategy to suppress the aggregation of fullerene molecules and enhance the long-term stability of films. Meanwhile, the introduction of flexible functional groups on the fullerene cage can not only increase the solubility and film uniformity but also improve the defect passivation effect [31,32]. Furthermore, flexible functional groups may also increase the physical entanglement between fullerene molecules beyond previously reported chemical interactions, which may enhance the stability of fullerene ETLs. Last but not least, the stable fullerene ETL also plays an important role in inhibiting the oxidation of Sn^2+^, which could effectively improve the long-term stability of TPSCs [33]. Thus, it is very important to explore the effect of fullerene materials containing flexible functional groups on the performance of TPSCs.

In this study, we synthesized a fullerene derivative with a flexible alkyl chain, PCBH, and investigated the influence of the functional group on the device performance of TPSCs. The long alkyl chain effectively boosted the solubility of PCBH in chlorobenzene and enhanced the interface contact of fullerene/perovskite, thus facilitating the electron extraction at the interface of fullerene/perovskite. As a result, the PCBH-based device yielded a maximum efficiency of 9.21%, which was much higher than that of the PCBM-based device (7.54%). More importantly, the long flexible chain effectively promoted the improvement of the quality and stability of the PCBH-based film, and the improved fullerene film stability efficiently suppressed the oxidation of Sn^2+^ in its underlying perovskite. Notably, this was markedly different from our previous report on the effect of the fullerene derivative PCBEH for LPSC [32]. Therefore, the PCBH-based device exhibited significantly enhanced stability. These results clearly suggest that regulating the functional group of the fullerene ETL is an effective strategy to improve the performance of tin-based devices.

## 2. Experimental Methods

### 2.1. Materials

All reagents and solvents were purchased from commercial suppliers and used as received. FAI, phenethylammonium iodide (PEAI), *N*,*N*-dimethylformamide (DMF), SnI_2_, dimethyl sulfoxide (DMSO), SnF_2_, and NH_4_SCN were bought from Sigma-Aldrich (St. Louis, MO, USA). PCBM (99%) was bought from Nano-C. PCBH was prepared according to a reported methodology [32]. After compounds were synthesized, they were purified by silica gel column chromatography to ensure the purity required for the device fabrication. The final products were characterized by ^1^H NMR, ^13^C NMR, and APCI-MS.

### 2.2. Device Fabrication

The TPSCs with a configuration of ITO/PEDOT:PSS/PEA_0.15_FA_0.85_SnI_3_/PCBM (or PCBH)/BCP/Ag) were prepared on ITO-based substrates. The patterned ITO-based substrates were ultrasonically cleaned with detergent, deionized water, and isopropanol, respectively. Next, the dried substrates were finally treated in an oxygen plasma for 5 min. The PEDOT:PSS (Clevios PVP AI4083, Hanau, Germany) layers were prepared by spin coating at 4000 rpm for 40 s, and baked at 130 °C for 15 min in ambient air. After cooling to room temperature, the substrates were transferred into a N_2_ filled glovebox. The PEA_0.15_FA_0.85_SnI_3_ perovskite layers were fabricated according to a previous work [34]. In total, 2 wt% PCBH and PCBM were dissolved in chlorobenzene and spin-coated onto the perovskite layers at 2000 rpm for 30 s. Next, the 0.5 mg/mL BCP solutions were spin-coated at 5000 rpm for 30 s and annealed at 70 °C for 5 min. Finally, an Ag electrode (100 nm) was formed by thermal evaporation through a shadow mask.

### 2.3. Characterization

The NMR experiments were performed on a Bruker Avance III 600 MHz NMR spectrometer (Billerica, MA, USA). Mass spectrometry (MS) was carried out on a Bruker Esquire HCT mass spectrometer with an atmospheric pressure chemical ionization (APCI) ion source in negative ion mode (APCI-MS). UV–Vis spectra were conducted using an ultraviolet-visible spectrophotometer (UV-2600). Cyclic voltammetry (CV) experiments were carried out under an Argon atmosphere at room temperature using a CH Instrument Potentiostat. A one-compartment cell with a standard three-electrode setup was used, consisting of a 1 mm diameter glassy carbon disk as the working electrode, a platinum wire as the counter electrode and a silver wire as the pseudo-reference electrode. Space-charge limited current (SCLC) was measured using a CHI660E electrochemical workstation in the dark. The current–voltage (*J*-*V*) curves were measured under one-sun illumination using a Keithley 2400 and Enli. Tec. AAA grade solar simulator. All devices were measured with a black metal mask (0.12 cm^2^). IPCE was calculated from the Enli QE-R equipment (Kaohsiung, Taiwan). The contact angles of the films were measured on a Rameé–Hart model 250 goniometer. SEM images were obtained on a field-emission scanning electron microscope (JEOL JSM-7610F plus, Akishima City, Tokyo, Japan). Steady-state photoluminescence (PL) experiments were conducted on a fluorescence quantum efficiency test instrument (Xipu Optoelectronics Technology Co., Ltd., Guangzhou, Guangdong, China).

## 3. Results and Discussion

To balance the electron transportation and the physical entanglement between the fullerene molecules [35], we designed the fullerene derivative PCBH (Scheme S1). We believed that the long alkyl chain would enhance the physical entanglement between PCBH molecules and elevate the stability of the film. At the same time, the long alkyl chain was expected to improve the solubility of the PCBH, thus increasing the penetration into the perovskite grain boundary during the spin coating process and enhancing the passivation effect on the perovskite defects [32,36]. Furthermore, the size of the hexyl chain was not too large, and was not expected to affect the molecular packing and charge transport in the corresponding fullerene-based films.

**Figure 1:**
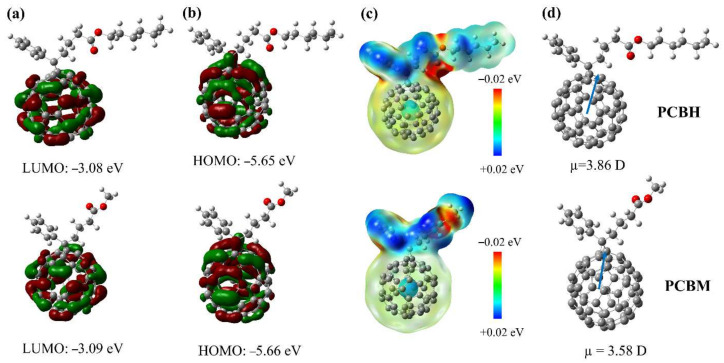
Electron density distributions determined by density functional theory (DFT) calculation for PCBH and PCBM. (**a**) LUMO, (**b**) HOMO, (**c**) electrostatic potential (ESP) results, and (**d**) dipole moments.

To explore the potential of PCBH to work as ETL in TPSCs, the frontier molecular orbital (FMO) energy levels, electrostatic potential (ESP) distributions and dipole moments were first investigated by first-principles calculation using density functional theory (DFT). As shown in Figure 1a,b, the PCBH and PCBM showed similar LUMO and HOMO distributions and values. The similar energy levels indicate that PCBH has a suitable energy level to serve as ETL in the TPSCs. Furthermore, as shown in Figure 1c, the ESP simulation illustrated the electron cloud distribution of PCBM and PCBH. Obviously, the two molecules featured similar negative charge distributions, which can be attributed to the same ester groups. In addition, as shown in Figure 1d, the introduction of the long alkyl chain increased the dipole moment of PCBH, which indicates that it has a larger dielectric constant. Therefore, the elevated dipole moment is expected to enhance the interface contact between PCBH and perovskite layers, as well as the mobility of the film [35].

**Figure 2:**
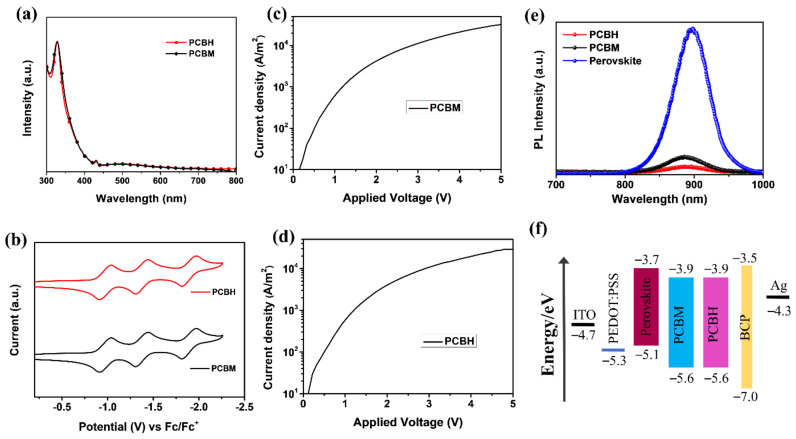
(**a**) UV–Vis absorption spectra of PCBH and PCBM in toluene solution. (**b**) Cyclic voltammetry waves for fullerene materials (PCBH and PCBM). *J*-*V* curves of the (**c**) PCBM- and (**d**) PCBH-based films for electron-only devices. Devices with a structure of ITO/Al/PCBH (or PCBM)/Al were fabricated. (**e**) Steady-state PL spectra of different perovskite films. (**f**) Energy levels of the corresponding layers applied in TPSCs.

PCBH was synthesized following a previously reported procedure [32]. The detailed synthetic route is shown in Scheme S1. The chemical structures of all the PCBH-related materials were confirmed using ^1^H NMR, ^13^C NMR spectrometry, and APCI-MS (Figures S1–S4). The UV–Vis spectrum was recorded to explore the optical properties of PCBH. As shown in Figure 2a, the maximum absorption onsets of the PCBH and PCBM molecules were observed at around 720 nm and 718 nm, respectively. Thus, the optical bandgaps of PCBH and PCBM were 1.72 and 1.73 eV, respectively (Table 1). The LUMO of PCBM and PCBH were obtained via cyclic voltammetry (CV) using Fc/Fc^+^ as a standard. As shown in Figure 2b, both exhibited three well-defined reversible reduction waves. The reduction functional wave onset of PCBH was nearly the same as that of PCBM, thus PCBH and PCBM possessed similar LUMO energy levels of −3.89 eV and −3.90 eV, respectively, which is consistent with the DFT calculation results. According to the results from the UV–Vis spectra and CV waves, the HOMO of PCBH and PCBM were calculated to be −5.61 eV and −5.63 eV, respectively.

The space-charge-limited current (SCLC) method was used to investigate the influence of the hexyl group on the electron mobility. Electron-only devices with a structure of ITO/Al/fullerene derivative/Al were fabricated. As shown in Figure 2c,d, the electron mobilities of PCBH and PCBM displayed a slight difference, being 4.68 × 10^−4^ and 4.62 × 10^−4^ cm^2^ V^−1^ s^−1^, respectively. Therefore, the PCBH showed comparable LUMO and HOMO energy levels, as well as electron mobility to that of PCBM, indicating that it may serve as an efficient ETL in TPSCs. A steady-state photoluminescence (PL) experiment was also applied to explore the influence of the hexyl group on the carrier dynamics. As shown in Figure 2e, the steady-state PL spectra of the perovskite, perovskite/PCBM, and perovskite/PCBH thin films were measured on glass substrates. The PCBM- and PCBH-coated perovskite films showed obviously quenched PL intensity compared to the pristine perovskite film. Interestingly, the PCBH-coated perovskite film displayed a much stronger PL-quenching effect than the PCBM-coated perovskite film, which can be attributed to the more efficient electron extraction and transport from perovskite to PCBH layer [9,37]. This result can also be attributed to the increased solubility of PCBH, which helps its molecules penetrate into the perovskite grain boundaries [32,38]. The energy levels of the corresponding layers applied in the TPSCs is shown in Figure 2f.

**Figure 3:**
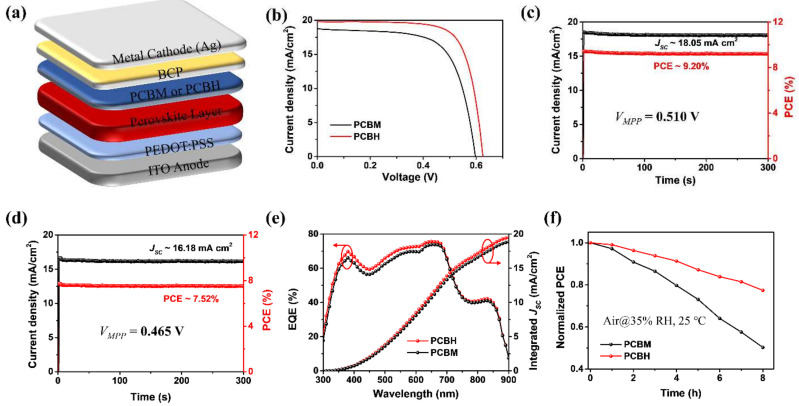
(**a**) Device architecture of the TPSCs. (**b**) The *J*–*V* curves of the best PCBH- and PCBM-based TPSCs. Maximal steady-state output for (**c**) PCBH- and (**d**) PCBM-based TPSCs and their corresponding power output. (**e**) The EQE and integrated current curves of the PCBH- and PCBM-based TPSCs. (**f**) The stability test of the PCBM- and PCBH-based TPSCs.

To demonstrate the effects of PCBH as the ETL in TPSCs, devices with a configuration of ITO/PEDOT:PSS/PEA_0.15_FA_0.85_SnI_3_/PCBH (or PCBM)/BCP/Ag were fabricated (Figure 3a) [34]. The flow chart of device fabrication process was provided in Figure S6. The photocurrent density-voltage (*J*-*V*) curves of the devices were provided in Figure 3b, and the corresponding key parameters of the TPSCs were displayed in Table 2. The device with PCBM ETL yielded an efficiency of 7.54%, which is comparable to the efficiency reported in a previous study [34].By contrast, the PCBH-based device provided an obviously improved efficiency of 9.21%, which is consistent with PCE statistic diagrams of PCBM- and PCBH-based TPSCs (Figure S7). The improved device performance could be attributed to the efficient electron extraction and passivation effect at the interface of PCBH/perovskite [39,40], which can reduce the carrier accumulation at the interface of ETL/perovskite in TPSCs.

For comparison, we provided a stable power output of the PCBM- and PCBH-based TPSCs. As displayed in Figure 3c,d, the improved efficiency and power output of PCBH-based devices suggest that the functional group of the fullerene derivatives is critical to the device’s performance. The incident photon-to-electron conversion efficiency (IPCE) spectra and the integrated photocurrent of the PCBH- and PCBM-based TPSCs are shown in Figure 3e. It is clear that the IPCE value of the PCBH-based device was slightly higher than that of the PCBM. The calculated current densities of 18.87 mA/cm^2^ and 19.60 mA/cm^2^ for the PCBM- and PCBH-based devices, respectively, were close to the values from the *J*–*V* curves. These results indicate that the PCBH-based devices yielded a better incident photon-to-current conversion efficiency.

Additionally, the stabilities of the unencapsulated TPSCs with PCBM and PCBH as ETLs are also provided. As shown in Figure 3f, the PCE of the PCBM-based device maintained less than 50% of its initial efficiency for only shelf-storage for 8 h at 35% humidity in air. By comparison, the PCBH-based device showed a loss of about 20% under the same conditions. This enhanced stability benefited from a denser and more stable PCBH layer, which effectively suppresses oxygen and moisture permeation [41].

**Figure 4:**
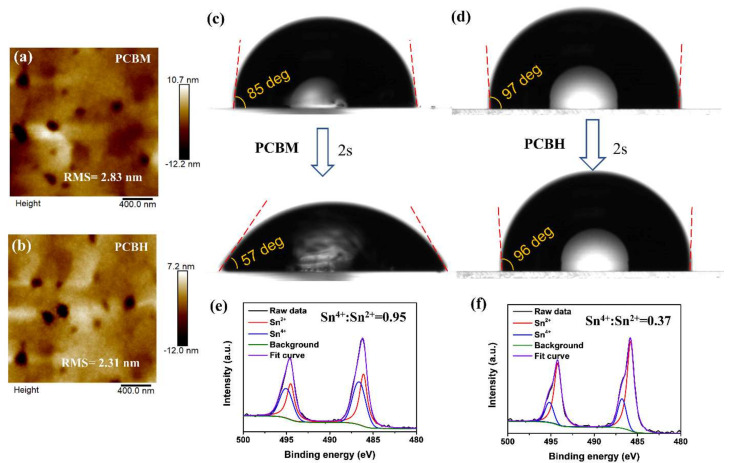
AFM images of the different fullerene films on perovskite substrates: (**a**) PCBM, (**b**) PCBH. The evolution of the contact angle measurement for the (**c**) PCBM and (**d**) PCBH. XPS Sn 3d spectra of the perovskite films with the upper layer of (**e**) PCBM and (**f**) PCBH removed. Atomic ratio of the Sn^4+^/Sn^2+^ derived from the XPS Sn 3d spectra.

As mentioned above, the hexyl group of PCBH significantly improved its solubility and solution processing. The enhanced solubility of PCBH (58 mg/mL compared to PCBM 46 mg/mL) is crucial to device fabrication, since the molecular structure and solubility of fullerene derivative have an important influence on the uniformity and stability of the film [29,30,31]. Firstly, AFM was used to further investigate the morphologies of the fullerene derivative films. As shown in Figure 4a,b, the PCBH film exhibited a surface root mean square (rms) roughness of 2.31 nm. By contrast, the PCBM-based film showed a roughness of 2.83 nm, which is much higher than that of the PCBH. The lower roughness may have been due to the better film-forming properties of PCBH, which could lead to denser PCBH films [10]. To verify this, the contact angles of the PCBM- and PCBH-based films were studied. As shown in Figure 4c,d, it was observed that water droplets on the PCBH layer showed a higher contact angle, of 97°, which can be attributed to its extended alkyl chain. By contrast, the contact angle value for the PCBM layer was 85°. Furthermore, we explored the time evolution of the contact angle of the PCBM- and PCBH-based films. It can be seen that the contact angle of the PCBH film showed a slight change after 2 s dripping, while the contact angle of the PCBM film displayed a significant decline during the same procedure. The higher water resistance stability of the PCBH film may be attributed to the physical entanglement of the extended flexible alkyl chain, which leads to a denser film. The denser film of PCBH may have increased the electron transport and collection efficiency [35], as well as suppressing the potential of oxygen injection [42,43]. Therefore, we recorded the XPS Sn 3d spectra of the different perovskite films with the upper fullerene layer removed. It should be noted that the PCBM and PCBH layers were removed after their coated perovskite films were stored in air for 2 h, and the obtained perovskite films were stored in nitrogen for XPS measurements. As shown in Figure 4e,f, the atomic ratio of the Sn^4+^/Sn^2+^ of the PCBM- and PCBH-coated perovskite were 0.95 and 0.37, respectively. The lower ratio of Sn^4+^/Sn^2+^ of PCBH suggests that the PCBH film suppressed the oxidation of Sn^2+^ [33], which is consistent with the above results. Therefore, the more homogenous and hydrophobic PCBH film supports the fact that PCBH-based devices showed higher stability than PCBM-based devices. These results clearly suggest that regulating the functional group of the fullerene molecules is also an effective strategy to improve the stability of their films and devices.

## 4. Conclusions

We synthesized a fullerene derivative, PCBH, with a flexible hexyl group and demonstrated it as an efficient ETL in TPSCs. The PCBH showed enhanced solubility, improved film morphology, and elevated electron extraction capability. As a result, the PCBH-based device yielded a higher PCE, of 9.21%, than a PCBM-based device (7.54%). Moreover, the PCBH-based film with boosted stability effectively suppressed oxygen and moisture permeation, which effectively enhanced the device’s stability. This study demonstrates that fullerene derivatives with extended flexible alkyl chains can significantly influence device efficiency and stability. It also provides a new direction for designing functional materials for efficient and stable TPSCs.

## Figures and Tables

**Table 1:** Summary data of CV and UV–Vis analysis for PCBH and PCBM.

Fullerenes	λ_abs_ (nm)	E_g_ (eV)	$E_{red}^{on}(V)$	LUMO (eV)	HOMO (eV)
**PCBH**	720	1.72	−0.91	−3.89	−5.61
**PCBM**	718	1.73	−0.90	−3.90	−5.63

**Table 2:** Summary of device performance analysis in Figure 3. The calculated Jsc values were obtained from the EQE curves.

Type	*V*_oc_ (V)	*J*_sc_ (mA/cm^2^)	Calculated *J*_sc_ (mA/cm^2^)	FF (%)	PCE (%)
PCBH	0.63	19.77	19.60	73.96	9.21
PCBM	0.60	18.74	18.87	67.40	7.54

## Data Availability

The data presented in this study are available on a reasonable request from the corresponding author.

## Supplementary Materials

The following supporting information can be downloaded at: https://www.mdpi.com/article/10.3390/nano12030532/s1. Scheme S1: The synthetic route of PCBH. Figure S1: ^1^H-NMR (600 MHz; CDCl3, 298 K) of T1. Figure S2: ^13^C-NMR (150 MHz; CDCl3, 298 K) of T1. Figure S3: APCI-MS of PCBH. Figure S4: ^1^H-NMR (600 MHz; CDCl3, 298 K) of PCBH. Figure S5: ^13^C-NMR (150 MHz; CDCl3, 298 K) of PCBH. Figure S6: The flowchart of the device fabrication process. Figure S7: PCE statistic diagrams of PCBM and PCBH-based TPSCs.
