# Supplementary material for: Fullerene Derivative with Flexible Alkyl Chain for Efficient Tin-Based Perovskite Solar Cells
Source: Nanomaterials (Basel). 2022 Feb 3;12(3):532. doi: 10.3390/nano12030532 (PMC8839661; doi:10.3390/nano12030532)
Supplement: Supplementary file 1 [file nanomaterials-12-00532-s001.zip › nanomaterials-1573494-supplementary.pdf]

nanomaterials

Supplementary Materials

Fullerene Derivative with Flexible Alkyl Chain for Efficient Tin-Based Perovskite Solar Cells

Chengbo Tian *, Chao Sun, Jingfu Chen, Peiquan Song, Enlong Hou, Peng Xu, Yuming Liang, Panpan Yang, Jiefeng Luo, Liqiang Xie and Zhanhua Wei *

Xiamen Key Laboratory of Optoelectronic Materials and Advanced Manufacturing, Institute of Luminescent Materials and Information Displays, Department of Materials, College of Materials Science & Engineering, Huaqiao University, Xiamen 361021, China; sunchao@stu.hqu.edu.cn (C.S.); chenjf@stu.hqu.edu.cn (J.C.); reinhardtspq@stu.hqu.edu.cn (P.S.); hquhel@163.com (E.H.); xupengpatrick@stu.hqu.edu.cn (P.X.); lymym@foxmai.com (Y.L.); panpanyang@stu.hqu.edu.cn (P.Y.); jfluo@stu.hqu.edu.cn (J.L.); lqxie@hqu.edu.cn (L.X.)

* Correspondence: cbtian@hqu.edu.cn (C.T.); weizhanhua@hqu.edu.cn (Z.W.)

Citation: Tian, C.; Sun, C.; Chen, J.; Song, P.; Hou, E.; Xu, P.; Liang, Y.; Yang, P.; Luo, J.; Xie, L.; et al. Fullerene Derivative with Flexible Alkyl Chain for Efficient Tin-Based Perovskite Solar Cells. *Nanomaterials* **2022**, *12*, 532. https://doi.org/10.3390/nano12030532

Academic Editor: Iván Mora-Seró

Received: 12 January 2022
Accepted: 31 January 2022
Published: 3 February 2022

Publisher's Note: MDPI stays neutral with regard to jurisdictional claims in published maps and institutional affiliations.

Copyright: © 2022 by the authors. Licensee MDPI, Basel, Switzerland. This article is an open access article distributed under the terms and conditions of the Creative Commons Attribution (CC BY) license (https://creativecommons.org/licenses/by/4.0/).

Synthesis of PCBH.

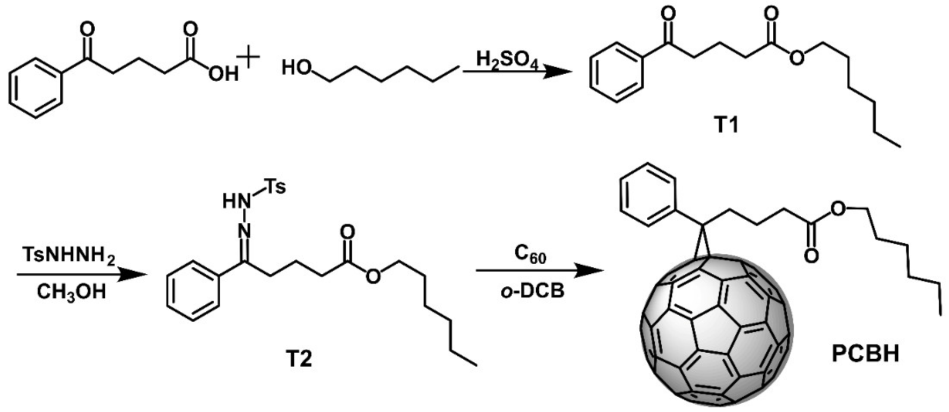

Scheme 1. The synthetic route of PCBH.

PCBH was synthesized following the methodology developed by Hummelen et al. for the preparation of PCBM [1].

Synthesis of T1. A mixture of 4-benzoylbutyric acid (1.92 g, 10 mmol), 1-hexanol (40 ml), and a catalytic amount of concentrated sulfuric acid was heated at reflux for overnight. Yield: 90% of a colorless oil.

Synthesis of T2. A mixture of T1 (2.76 g, 10 mmol), p-toluenesulfonylhydrazide (2.23 g, 12mmol), methanol (60 ml), and a catalytic amount of concentrated hydrochloric acid was heated at reflux for overnight. Afterwards it was cooled to room temperature and kept overnight in a refrigerator for crystallization of the product. The product was collected by filtration, washed with cold methanol and dried in air to yield tosylhydrazone as a white crystal (4.05 g, 90%).

Synthesis of PCBH. A mixture of T2 (0.89 g, 2 mmol) and NaOMe (0.13 g, 2.4 mmol) in dry pyridine (15 ml) was stirred under N_2 atmosphere at room temperature. After stirring for 15 min, the reaction mixture was mixed with a solution of C_{60} (1.44 g, 2 mmol) in o-dichlorobenzene (80 mL) and stirred for 20 h at 80 °C. Afterwards it was heated to reflux overnight. After cooling to room temperature, the reaction mixture was

concentrated in vacuo. The crude product was purified by silica gel column chromatography to obtain the product (0.69 g, 35%).

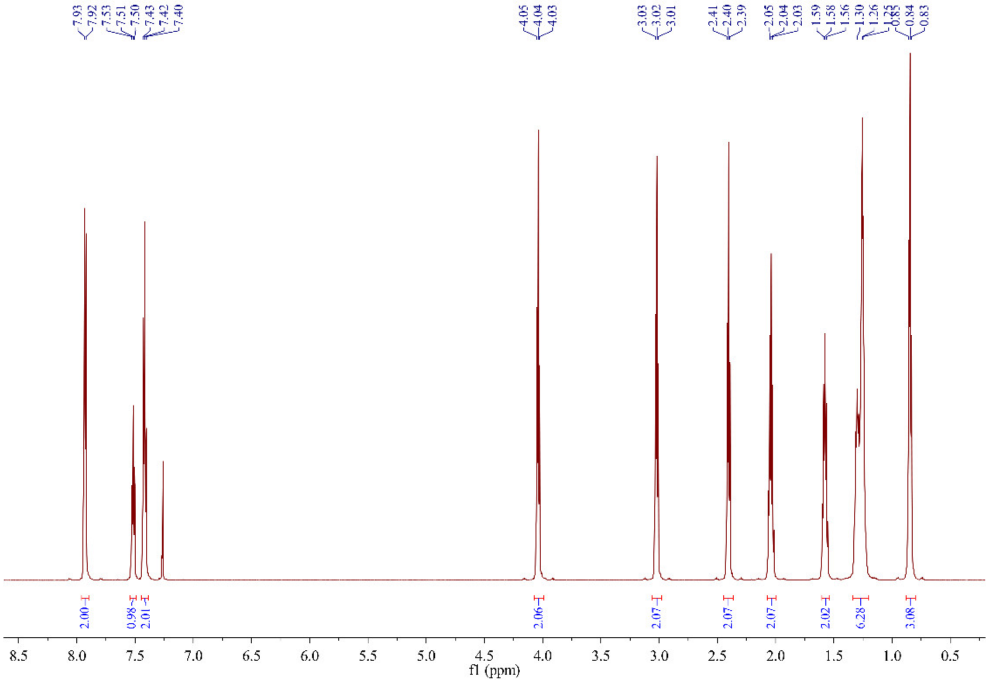

Figure S1. ^{1}H-NMR (600 MHz; $CDCl_3$, 298 K) of T1.

^{1}H NMR (600 MHz; CDCl3, 298 K) δ 7.93 (d, J = 8.0 Hz, 1H), 7.51 (t, J = 6.9 Hz, 1H), 7.42 (t, J = 7.5 Hz, 1H), 4.04 (t, J = 6.6 Hz, 1H), 3.02 (t, J = 7.1 Hz, 1H), 2.40 (t, J = 7.1 Hz, 1H), 2.04 (p, J = 7.2 Hz, 1H), 1.64 – 1.51 (m, 1H), 1.36 – 1.18 (m, 3H), 0.84 (t, J = 6.6 Hz, 2H).

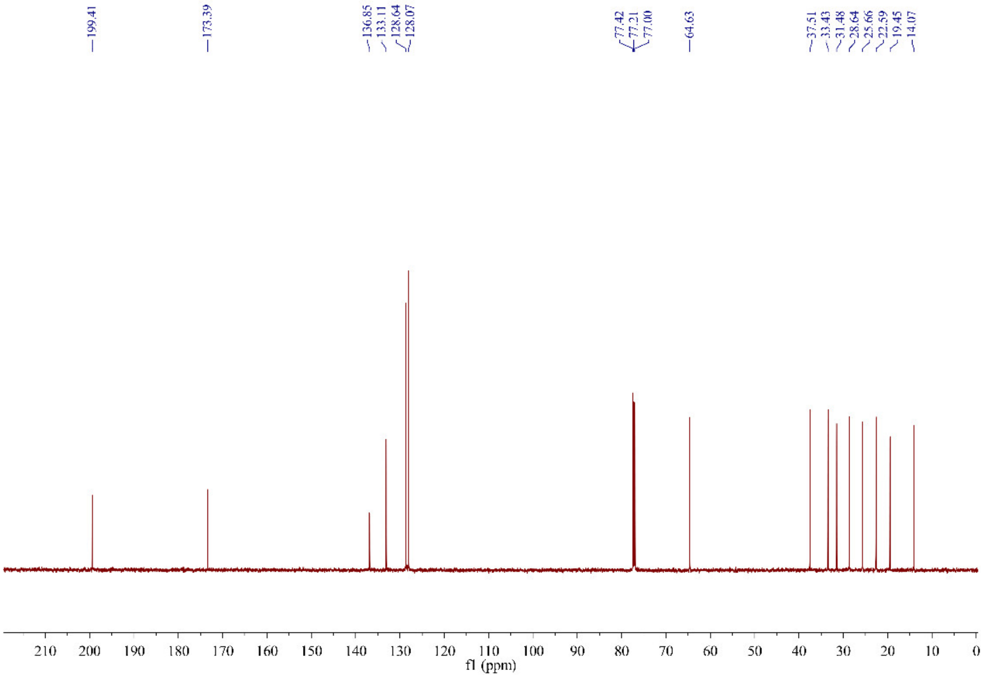

Figure S2. ^{13}C-NMR (150 MHz; $CDCl_3$, 298 K) of T1.

^{13}C NMR (150 MHz, CDCl3, 298 K) δ 199.41, 173.39, 136.85, 133.11, 128.64, 128.07, 77.42, 77.21, 77.00, 64.63, 37.51, 33.43, 31.48, 28.64, 25.66, 22.59, 19.45, 14.07.

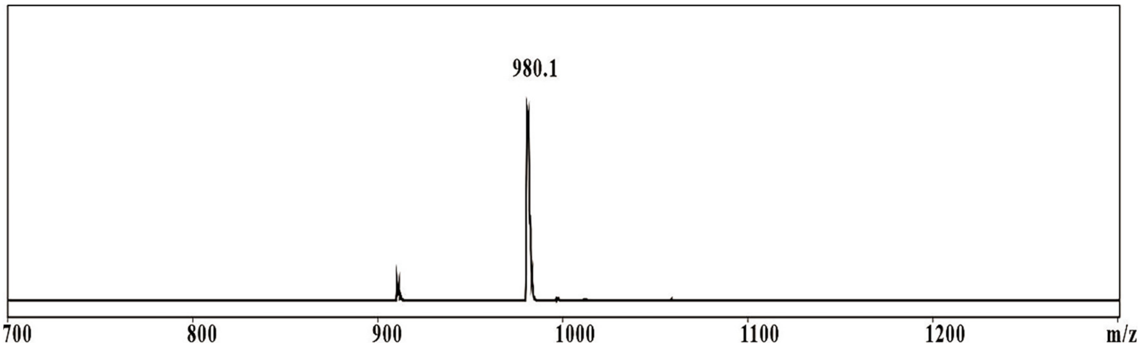

Figure S3. APCI-MS of PCBH.

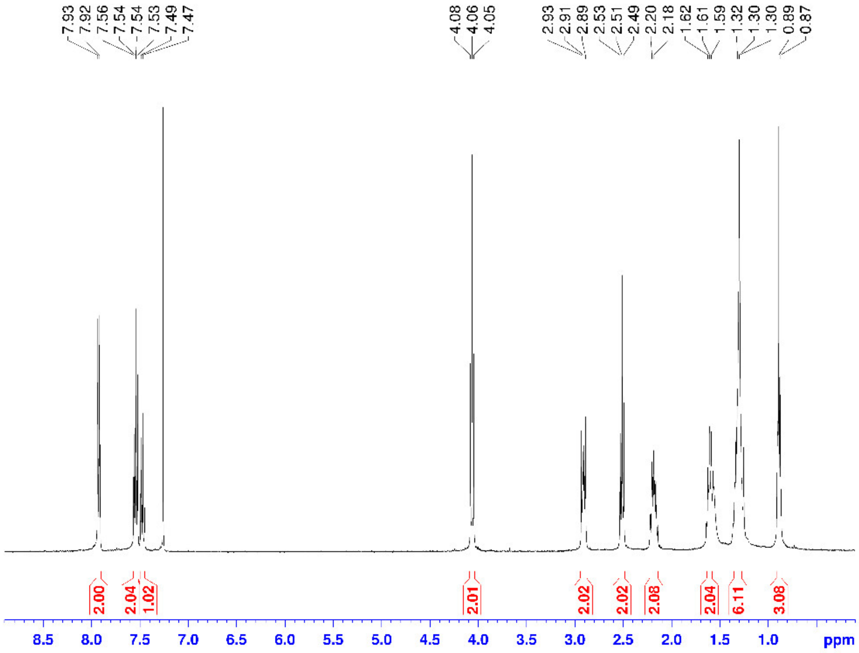

Figure S4. ^{1}H-NMR (600 MHz; $CDCl_3$, 298 K) of PCBH.

^{1}H NMR (600 MHz; CDCl3, 298 K) δ(ppm) 7.93 (d, J = 7.0 Hz, 2H), 7.54 (q, J = 4.9 Hz, 1H), 7.48 (d, J = 7.4 Hz, 2H). 4.06 (t, J = 6.8 Hz, 2H), 2.91 (t, J = 8.2 Hz, 2H), 2.51 (t, J = 7.5 Hz, 2H), 2.19 (d, J = 8.1 Hz, 2H). 1.61 (t, J = 7.3 Hz, 2H), 1.31 (m, 6H), 0.88 (t, J = 7.0 Hz, 3H).

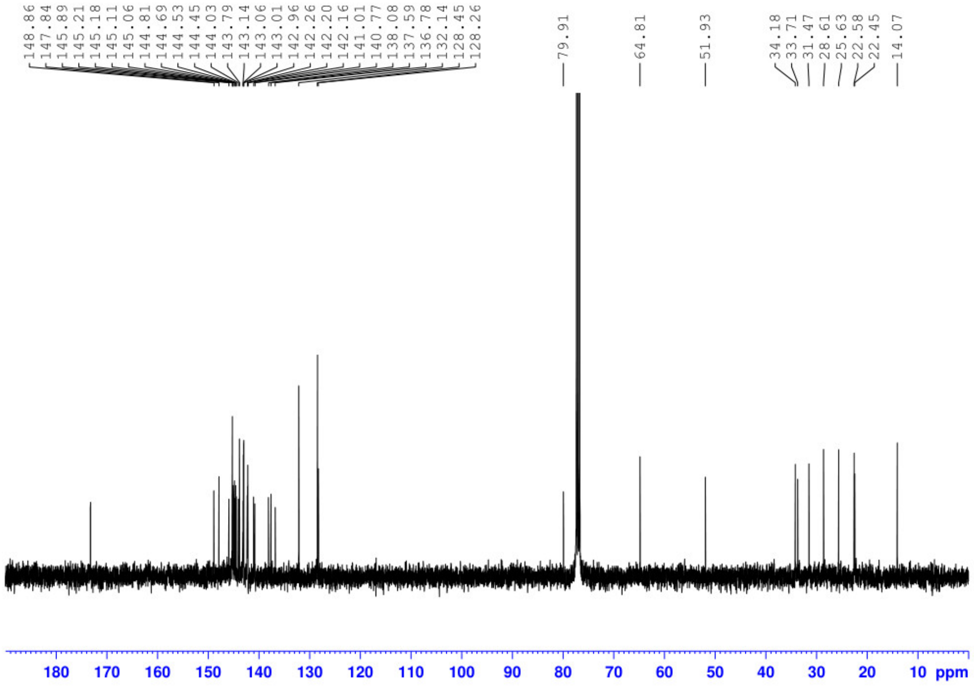

Figure S5. ^{13}C-NMR (150 MHz; $CDCl_3$, 298 K) of PCBH.

^{13}C NMR (150 MHz; $CDCl_3$, 298 K) δ (ppm) 14.07, 22.45, 22.58, 25.63, 28.61, 31.47, 33.71, 34.18, 51.93, 64.81, 79.91, 128.26, 128.45, 132.14, 136.78, 137.59, 138.08, 140.77, 141.01, 142.16, 142.20, 142.26, 142.96, 143.01, 143.06, 143.14, 143.79, 144.03, 144.45, 144.53, 144.69, 144.81, 145.06, 145.11, 145.18, 145.21, 145.89, 147.84, 148.86, 173.18.

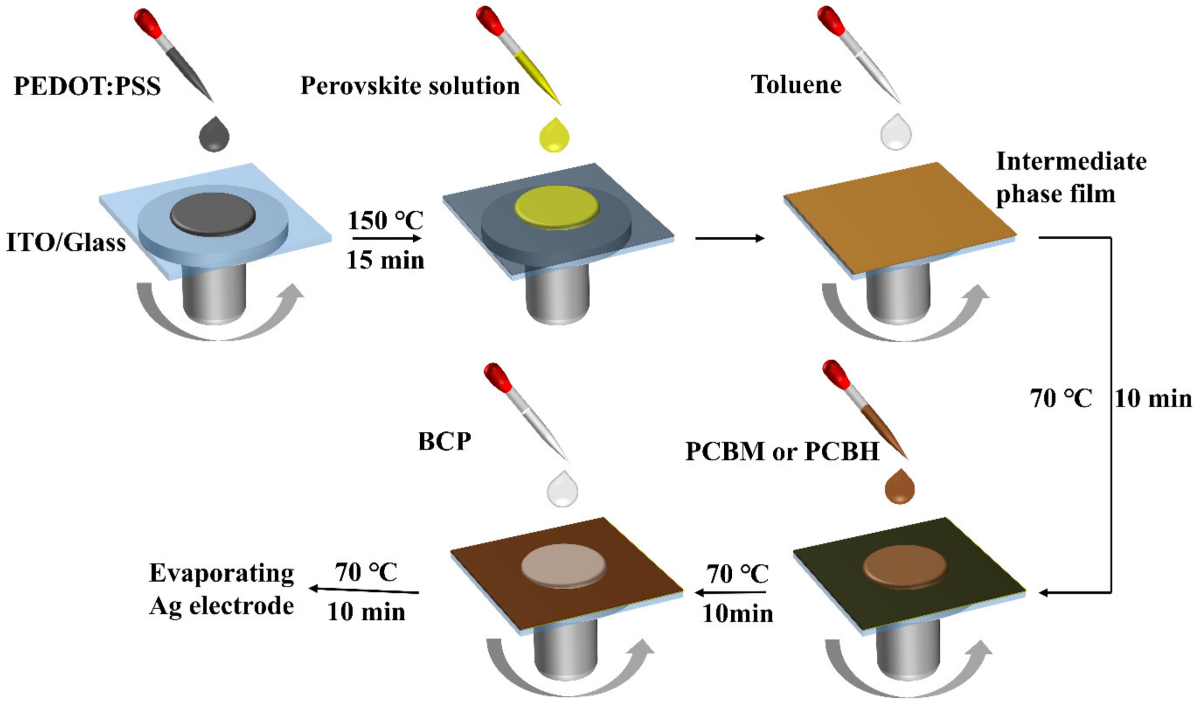

Figure S6. The flowchart of the device fabrication process.

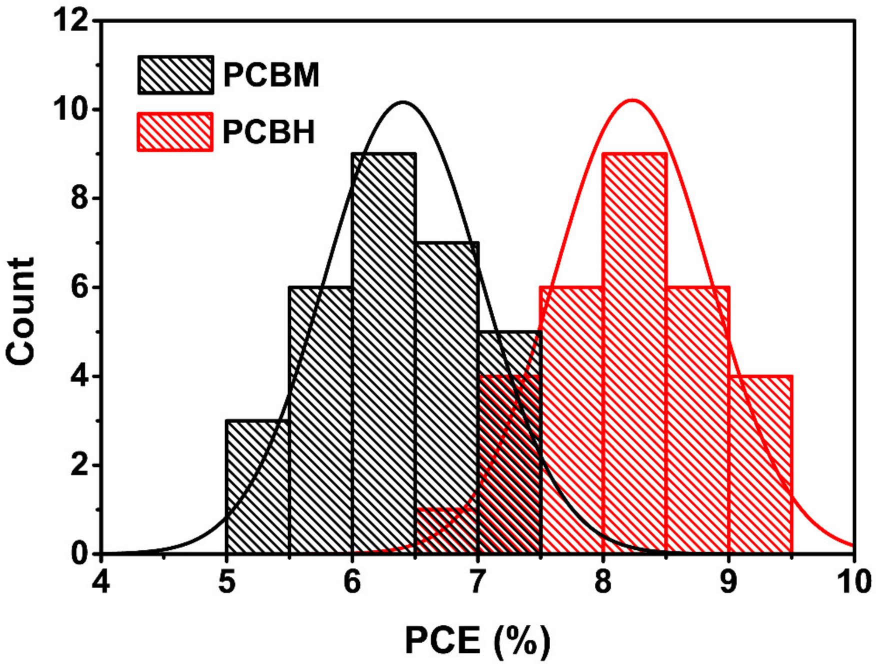

Figure S7. PCE statistic diagrams of PCBM and PCBH-based TPSCs.

Reference

1. Hummelen, J.C.; Knight, B.W.; Lepeq, F.; Wudl, F.; Yao, J.; Wilkins, C.L. Preparation and Characterization of Fulleroid and Methanofullerene Derivatives. *J. Org. Chem.* **1995**, *60*, 532–538.
